# Determining the Types of Contrasts: The Influences of Prosody on Pragmatic Inferences

**DOI:** 10.3389/fpsyg.2018.02110

**Published:** 2018-11-08

**Authors:** I-Hsuan Chen, Chu-Ren Huang, Stephen Politzer-Ahles

**Affiliations:** Department of Chinese and Bilingual Studies, The Hong Kong Polytechnic University, Kowloon, Hong Kong

**Keywords:** prosody, scalar inferences, numeral-classifier phrases, negative polarity items, intonation

## Abstract

This study explores the issues involving pragmatic inferences with prosodic cues. Although there is a well-established literature from multiple languages demonstrating how different pragmatic inferences can be applied to the same syntactic structure, few studies discuss whether prosody can determine types of alternative sets based on the same syntactic structure. In Mandarin Chinese, the same sentence containing a numeral-classifier phrase as a negative polarity item can be employed for two types of scalar inferences based on either the numeral or the noun. The sentence wo *yi zhi mayi dou mei kan dao* (“I didn’t even see one ant”) can induce two different scalar inferences: Quantity-contrast (‘I did not see one ant, much less two ants, three ants, and so on’ by drawing a contrast against the minimal quantity of one), and Type-contrast (‘I did not see an ant, much less a dog, a cat, a human being, and so on’ by drawing a contrast against the minimally surprising type, that of ants). Taking advantage of similar sentences with the syntactic structure and lexical items, our study examines whether prosodic conditions can guide people to choose pragmatic inferences from a set of options based on the same syntactic structure. The experiments of this study are designed to answer whether prosody interacts with contextual information in this grammatical structure. The results suggest that Mandarin speakers can use sentence prosody to determine which inference is intended, at least in experimental contexts that directly probe explicit awareness of prosody. Prosody does play a role in inducing scalar inferences, but contextual information can override the effects of prosody. Each prosodic pattern can evoke a specific set of scalar inferences, but quantity-contrast inferences are favored over type-contrast inferences. Our experiments show that prosodic prominence can serve as a linguistic cue to pragmatic inferences.

## Introduction

Pragmatics is the study of how signs are used and interpreted in context by language users and their interlocutors (Morris, 1938). The studies of pragmatics focus on the context-dependent meanings which are systematically abstracted from the logical form or the content of a construction concerned in syntax and semantics (Grice, 1989; Horn and Ward, 2005). In order to interpret information from a speaker, the hearer has to take the interaction of grammatical structure and context into consideration. The scalar inferences discussed in this study are cases showing that the hearer evokes a mental scalar model from a grammatical construction and context (Fillmore et al., 1988). The inferences from a scalar model compare the possibility of all alternatives on a defined scale.

This study investigates whether prosody influences pragmatic inferences by examining the types of inferences inferred from negative polarity items (NPIs) in Chinese. As a tonal language, Chinese has both syllable-level lexical tones and sentence-level intonation. The syllable-level lexical tones have been described as “small ripples riding on large waves of intonation” (Chao, 1933). Intonation interacts with syllabic tones without canceling their acoustic effects. The prominence of intonation is regarded as expanding pitch range. For example, prominent words have larger pitch range, longer duration, and higher intensity in prosody (Shih, 1988). Particularly, contextually focused words in a sentence are prominent in pitch height and intensity (Yuan, 2004). The present study examines how sentence-level intonation, particularly focus, influences the interpretation of NPIs.

Negative polarity items are expressions that are only grammatical under certain semantic contexts, such as negation and other forms of downward entailing contexts (Giannakidou, 2011; Israel, 2011). For example, in English, *I haven’t ever been to France* is grammatical but ^∗^*I have ever been to France* is not; *ever* is an NPI which is only grammatical in NPI-licensing contexts. NPIs have been observed across many languages (Haspelmath, 1997). They are often words referring to very small amounts, e.g., *I didn’t sleep a wink, He won’t spend a red cent, They don’t give a rat’s ass about this topic*. In such cases, the negation of such a small amount allows the hearer to infer that larger amounts are also not true: e.g., if somebody did not sleep “a wink” then they surely did not sleep for a long time either. These types of small-quantity expressions which occur in environments related to negation are called *minimizers*, and are a type of NPI. Across languages, minimizers are widely employed for pragmatic emphasis, due to their robust scalar inferences (Giannakidou, 2011; Israel, 2011). Minimizers induce scalar reasoning because they evoke a mental scalar model with all the alternatives ranked for contrasting (Israel, 2011). Since minimizers refer to an endpoint of a scale, they can contrast with all the other alternatives along the scale for emphasis (Fauconnier, 1975; Horn, 1989). That is to say, if the smallest or weakest item on the scale (e.g., sleeping a wink) is not true, then all larger or stronger items (sleeping a minute, sleeping an hour, etc.) must also not be true.

The paper reports three experiments regarding scalar implicatures and prosody. In each experiment in this study, all the participants provided their informed consent before they began the survey. Each experiment had both a traditional character version and a simplified character version. The traditional character version was distributed in Taiwan and Hong Kong, while the simplified character version was distributed in Mainland China. When the survey was advertised through the platforms of social media, both links were provided and volunteers could choose based on their preference.

In numeral-classifier languages such as Mandarin Chinese, ‘one’-phrases, which are composed of the numeral ‘one,’ a classifier or a measure word, and a noun, are pervasively used as minimizers, as in example (1) below. Specifically, sortal classifiers are employed for categorizing a semantically salient perceptual property of a noun which can be individuated (Ahrens and Huang, 2016).

(1) wo yi zhi cangying dou mei kandao
  I one CLF fly FOC NEG see
  ‘I did not see even one fly.’

Just as in the examples above, sentences with numeral-classifier phrases like (1) also elicit inferences about what the phrase is being contrasted with. (In this and other examples, CLF stands for classifiers, FOC for focus markers, and NEG for negation.) Specifically, for a sentence like (1), two types of inferences are possible. The sentence can infer that the speaker saw ‘not even one fly, much less two’ if the minimizer is interpreted as invoking a quantity-based contrast, while it can instead imply that the speaker saw ‘not even one fly, much less one human being’ if the minimizer is interpreted as invoking a type-based contrast. In the quantity-contrast interpretation, the minimal amount that is being invoked is “one,” and this is raised in contrast with greater amounts (“two flies,” and “three flies,” etc.); in the type-contrast interpretation, the minimal amount is some type of noun that has a high probability of occurring in this context. For example, this sentence is uttered in a context where there are likely to be flies, and this is raised in contrast with nouns that are even less likely or prototypical in this context. The quantity-contrast interpretation is straightforward due to the involvement of a numeral phrase, while the type-contrast interpretation is relatively less straightforward since it is relevant to the shared knowledge of the contexts. However, it is clear that the noun chosen for contrasting is the proposition which is assumed to be the most likely one.

In other numeral classifier languages such as Japanese and Korean, the distinction of the two types of inferences is reflected in morphology and word order (Lee, 2006; Nakanishi, 2006). However, in Mandarin, the two sets of inferences occur in the same word order, syntax, and semantics. Native Mandarin speakers thus require other cues to discern the pragmatic differences. It has been noted in studies of NPIs that minimizers are claimed to tend to occur in constructions that can attract people’s focus (Israel, 2011). For instance, an expression interpreted as a minimizer carries an emphasized intonation which is different from its other uses. In line with this observation, Mandarin minimizers tend to occur in the preverbal construction as in (1): this sentence has a Subject-Object-Verb word order, which differs from the Subject-Verb-Object word order that is canonical and unmarked in Mandarin. This preverbal position, where “one fly” occurs in sentence (1), has received substantial attention in the literature and has been regarded to carry focus (Zhang, 2000; Tsai, 2004; Huang et al., 2009). It is also noted that ‘one’-phrases may bear a different prosodic stress when they are used as minimizers as opposed to when they are used normally (Chao, 1968). According to these studies, a connection between prosodic stress, focus of attention, and pragmatics can be inferred. However, the issues of how focus is perceived by native speakers and of whether prosodic stress modulates the inferences drawn by speakers in this type of sentence have been barely touched upon.

On the other hand, scalar inferences have been shown to be associated with grammatical structures. For example, Chierchia (2004) and Chierchia et al. (2012) argued that a grammatical well-formedness condition based on pragmatics must be checked during the morphosemantic processing of NPIs and scalar implicatures. Other accounts differ on how or when grammatical information is integrated to process scalar references. However, to the best of our knowledge, the question of whether prosody would also be checked has not been answered. For instance, it is already known that prosody has an immediate impact on the incremental interpretation of an utterance that is unfolding: for example, prosodic focus influences how likely listeners are to commit to interpreting *some* as *not all* (Degen and Tanenhaus, 2015) and *or* as exclusive *or* (Chevallier et al., 2010), and to disambiguate the meaning of sentences with attachment ambiguities like *Tap the frog with the flower* (Snedeker and Trueswell, 2003). In the study of *some* as *not all*, the impact of prosody is whether to apply the inferences, while in the case of the attachment ambiguity the question is whether prosody can help to differentiate the actual differences in syntactic structure. However, it has not yet been empirically demonstrated that prosody has an impact on the inferences elicited by minimizers like those described above. The abovementioned examples are cases where ambiguity derives from the choice whether or not to realize an implicature at all, or the choice between different syntactic structures to build; on the other hand, the interpretational ambiguity in Mandarin minimizers comes from two types of alternative sets and not from syntactic differences or from the presence or absence of an implicature (as the same implicature is made under both interpretations, the implicature is simply applied over different alternative sets).

The experiments of this study are designed to answer this question. The experiments force participants to consider prosodic conditions by using identical, well-formed morphosyntactic structures. In particular, Chinese provides an interesting and challenging environment for testing the role of prosody in scalar inferences. The prosody of Chinese, a tonal language, is an overlaying pattern which modifies pitch ranges and intensities, instead of lexicalizing pitch patterns, as discussed above. Since Chinese prosody does not depend on change of pitch value *per se*, our experiment has the added value of being able to show that it is the linguistic concept of prosody that plays the central role in processing scalar inferences. In particular, the three experiments of the study attempt to show whether prosody interacts with contextual information in the processing of scalar inferences.

The critical stimuli of the three experiments in this study are sentences with the structure exemplified in (2). A prosodic stress is superimposed either on the numeral-classifier constituent or on the noun of the numeral phrase, as shown in the bolded sections. The stimuli were produced by a female native Mandarin speaker, who speaks only Beijing Mandarin without other dialects.

(2) (a) jintian maomi kafeiguan mei kai, **yi** **zhi** maomi dou mei you
  (b) jintian maomi kafeiguan mei kai, yi zhi **maomi** dou mei you
    today cat café NEG open one CLF cat FOC NEG exist
  ‘The cat café is closed today. There isn’t even one cat.’

Although other numeral-classifier languages such as Japanese can rely on morphology to distinguish the two types of scalar inferences, it has been noted that the elements attached by a scalar particle, such as the noun or the numeral-classifier unit of a numeral phrase, carry an emphatic prosody (Nakanishi, 2006). In the setting of a quantity contrast, the numeral-classifier unit is stressed; in the setting of a type contrast, the noun is stressed. Therefore, our intuition suggests that the prosody in (2)a should be more likely to evoke a quantity contrast (i.e., an interpretation like “I didn’t even see one cat, let alone two cats, three cats, etc.”), whereas the prosody in (2)b should be more likely to evoke a type contrast (i.e., an interpretation like “I didn’t even see one cat, let alone one person, one bird, etc.”). The purpose of the present study was to see whether this intuition is supported by empirical data from naïve listeners.

Each experiment has a different task for the participants to respond to the stimuli. In Experiment 1, the participants were asked to judge whether the sentence which they heard from an audio clip was consistent with a paragraph they read previously, which set up a context consistent with either a quantity contrast or a type contrast. The design of Experiment 2 is the same as that of Experiment 1, but the participants were asked to give consistency ratings on a Likert scale rather than binary judgments of consistency. In Experiment 3, the participants read a context and then heard two auditory versions of the sentence with different prosody, and were instructed to select the version that better fit the context. The three tasks were made to test whether prosody is a determinant of the types of scalar reasoning and how much Mandarin speakers are aware of prosody. The results can help to validate the associations of the unconnected pieces in the literature of focus, prosody, and pragmatic inferences.

## Experiments

We performed three experiments involving reading and listening to texts, with slightly different procedures, to test how participants evoke scalar implicatures based on the available information.

### Experiment 1: Matching Scalar Inferences

The first experiment is designed to test whether prosody would help Mandarin native speakers to determine types of scalar inferences when there is no distinction in the grammatical form. In the experiment, the participants had to read a short paragraph and to listen to a sentence. They had to judge whether the sentence they heard matched the provided context.

#### Participants

Sixty-nine native speakers of Mandarin (60 users of traditional Chinese characters and 9 users of simplified Chinese characters) were included in the first experiment. Two were removed from analysis because they did not correctly respond to baseline questions (see section “Procedure”), leaving 67 participants (aged 20–60, mean 31) in the final analysis.

#### Materials

The experimental stimuli comprised 12 sentences along with 16 fillers. A short paragraph was provided to set up the relevant context for each stimuli sentence, as shown in (3). The sentence always referred to some set that did not have some property. In (3), for example, the dog park does not have dogs, which should be expected to be most likely encountered in the defined setting. Another type of noun, human being, is involved as an alternative to be contrasted with dogs in this setting. Each context paragraph either indicated that the most likely property is not present but the other one is (e.g., the park did not have dogs but did have people, as in 3a), or that both properties are not present (e.g., the park had neither dogs nor people there). Finally, it introduced a speaker about to say the critical sentence.

(3) *Zhangwei dao le youmingde liugou gongyuan, pingchang zheli henduo gou.* ‘Zhangwei went to a famous dog park. Usually there were a lot of dogs.’
  (a) Jintian meiyou gou que you ren zai gongyuan li sanbu
    ‘Today there were no dogs, but there were people walking in the park.’ [Yes-context]
  (b) Jintain gongyuan li meiyou gou ye meiyou ren
    ‘Today there were neither dogs nor people in the park’ [No-context]
    *Zhangwei huilei hou gen ni shou:* ‘Zhangwei came back and told you:’

The experimental stimuli appeared in the syntactic format of (1). The context paragraph (3) was presented in Chinese characters, and the critical sentence (4) presented auditorily afterward:

(4) *Wo jiantian qu le liugou gongyuan*, ‘I went to the dog park today.’
  (a) **yi** **zhi** gou dou mei kandao
    one CLF dog FOC **NEG** see
  (b) yi zhi **gou** dou mei kandao
    one CLF dog FOC **NEG** see
    ‘I did not see even one dog.’

The two versions of the audio files both express the lack of a specific property which is the most expected in the defined set, e.g., the speaker did not see even one dog at the dog park. The only difference is that one has the prosodic stress on the numeral-classifier combination (4a), while the other stresses the noun (4b). For ease of reference, we refer to the former as quantifier stress, and the latter as noun stress. Based on the four conditions, the experiment followed a 2 × 2 design: PROSODIC STRESS (*noun stress* vs. *quantifier stress*) × CONTEXT (*type alternative present* vs. *type alternative absent*). The items were organized into four lists in a Latin square design.

The fillers can be divided into two groups. The first group contains six sentences which mismatch the content from the audio files. There are three types of mismatches including number, quantity, and location. One example of number mismatch is provided in (5), where the context and the critical sentence are unambiguously semantically inconsistent. The fillers both serve as a check that the received data are valid, and to distract participants from the experimental manipulation.

(5) Reading context: *Mama qie le san ge pingguo, danshi meiyou chi. Baba gen ni shuo:* ‘Mom cut three apples, but didn’t eat them. Dad told you:’
  Audio context: *Mama yi ge pingguo dou mei qie*. ‘Mom did not cut even one apple. ’

The other group of fillers consists of 10 sentences from another experiment for investigating the scalar implicatures from Mandarin *youxie* ‘some.’ The full list of stimuli is available at https://osf.io/nsgfv/.

#### Prediction

In the context where the type alternative is present (3)a, we expected that the critical sentence with prosodic focus on the noun, compared to the critical sentence with prosodic focus on the numeral and classifier, would be less consistent with the context. This is because prosodic focus on the noun (i.e., “I didn’t even see one *dog*”) should license the inference that the speaker didn’t see anything else either, including the type alternative (i.e., “I didn’t even see one *dog*, let alone one person”). Thus, we expected a difference in consistency ratings between the two prosodic conditions in this context. On the other hand, in the context where the type alternative is absent (3)b, we expected no difference in consistency ratings between the two prosodic conditions, since both inferences (i.e., “I didn’t even see one dog, let alone one person” and “I didn’t even see one dog, let alone two”) are consistent with the context in which there are neither dogs nor people in the park.

#### Procedure

This experiment was administered online via Ibex Farm (http://spellout.net/ibexfarm/). At the beginning of the experiment, participants indicated their consent to participate, provided demographic information (age, sex, and native language), and answered two questions about the experiment meant to probe whether they had read the instructions One question was which university the experiment was being run by, and the other question was how many trials there would be in the experiments. The 12 items along with 16 fillers were then randomly presented in a Latin square design after three practice trials. For each trial, participants read a short Mandarin paragraph which either established a context where a contrasting type is present (e.g., (3)a, in which there are no dogs but there are people), or a context where the contrasting type is absent (e.g., (3)b, where there are neither people nor dogs in the park). When they finished reading at their own pace, they then clicked a button to listen to a sentence relevant to this setting with either a stressed noun or a stressed numeral-classifier combination. The task for the participants was to judge whether what they heard could fit the context that they read. They were asked to click either *consistent* or *inconsistent* based on their own judgments. After submitting the answer, a participant could move onto the next question. The whole survey was self-paced. It took less than 30 min for the participants to finish the survey.

#### Results

**FIGURE 1:**
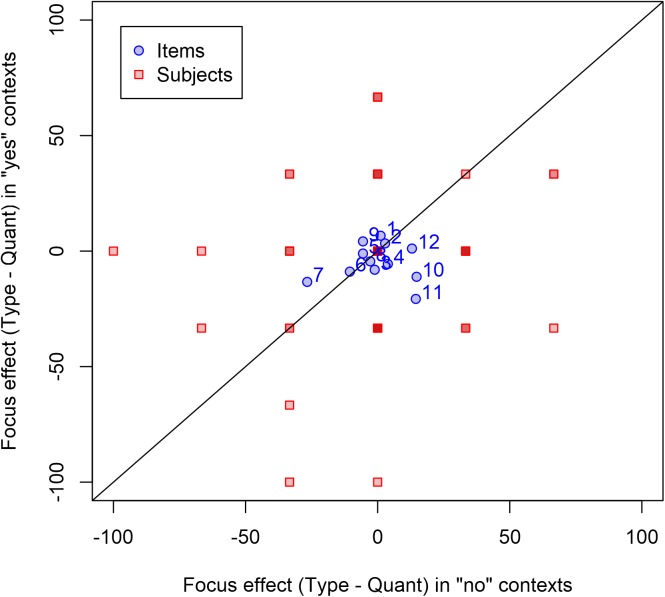
Effects of prosody in Experiment 1. Each point represents one stimulus item or one participant. The x-axis represents the difference in percentage acceptance for noun stress vs. quantifier stress prosody in contexts where the alternative referent is not present (i.e., when neither dogs nor people were in the park), such that negative values indicate when noun stress prosody was accepted less than quantifier stress prosody. The y-axis shows this same difference, but in contexts where the alternative referent is present (i.e., when there were no dogs in the park but there were people). Note that at several places there are multiple subjects with points in the same location; these can be recognized by the darker square backgrounds (since the background coloring is opaque). For clarity, point labels are provided only for items, not for subjects.

The full dataset and analysis code (for the R statistical programming environment) are available at https://osf.io/nsgfv/. Overall, in contexts where the type alternative referent was present, participants accepted 85.6% of sentences with quantifier stress and 82.1% of items with noun stress, a difference in the expected direction; also consistent with the predictions, they showed less difference in acceptance of different prosody in the context where the alternative referent is also not available, accepting 89.6% of sentences with quantifier stress and 90.0% of sentences with noun stress. Figure 1 shows the variability of the effect across items (by-subject aggregates are not plotted; since each participant only saw a small number of items and thus only had a small number of possible outcomes per condition [0, 33, 66, or 100%], there is little subject-wise variability to be seen). In Figure 1, because the prediction was that there would be a larger prosody effect (in the negative direction) in these context than in contexts where the alternative is available, this means that points below the diagonal represent items showing effects consistent with the prediction, and points above the diagonal are inconsistent with the prediction.

The results were statistically analyzed using generalized (binomial) mixed-effects models with crossed random effects for subjects and items (Baayen et al., 2008). The predictors PROSODIC STRESS (*noun stress* vs. *quantifier stress*) and CONTEXT (alternative type present vs. alternative type absent) were sum-coded (as 0.5 and -0.5) and used as fixed predictors, along with their interaction; random effects of these three parameters were also fit for items (Barr et al., 2013), but not for subjects, since each subject had too few trials to fit this complex structure well. The significance of the crucial PROSODIC STRESS ^∗^CONTEXT interaction was assessed with a log-likelihood test comparing this model to a maximally similar model without the fixed interaction effect. The interaction did not reach significance in this comparison [χ^2^(1) = 0.14, *p* = 0.707].

The results of the experiment showed a numerical trend in the predicted direction, such that prosody influenced sentence acceptability in contexts where the alternative type was present and less so in contexts where the alternative type was absent. However, this trend was not statistically significant. Furthermore, even in contexts where prosody should have elicited an inference that does not fit the context (i.e., “I didn’t even see one *dog* [let alone one person],” in a context where there were no people in the park), sentence acceptance was still quite high, over 80%; this suggests that participants were not influenced very much by prosody, as long as the lexico-semantic content of the sentence fit the context. For this reason, we attempted to conceptually replicate the experiment, while making changes to potentially increase the size of the effect. We suspected that the binary nature of the acceptability judgment may have forced participants to ‘accept’ sentences even when they were aware of slight inconsistencies; thus, in this experiment we instead had participants rate sentences on a six-point Likert scale, which we predicted might allow them to register their awareness of the prosodic mismatch and thus might increase the chances of observing a prosodic effect. Otherwise, the predictions for Experiment 2 are the same as for Experiment 1: we expect worse ratings for noun-stress prosody than for quantifier-stress prosody in contexts where the alternative type is present, but not in contexts where the alternative type is absent.

### Experiment 2: Rating Scalar Inferences

The procedure of the second experiment is the same as that of the first experiment, except that in this experiment participants had to rate to what extent the inferences from the audio contents were consistent with the provided contexts, rather than making a binary judgment. The predictions are the same as in Experiment 1.

#### Participants, Materials, and Procedure

Seventy-eight native speakers of Mandarin (63 users of traditional Chinese characters, 15 users of simplified Chinese characters) took part in this experiment. Ten were excluded for answering baseline questions incorrectly; the exclusion criteria and data collection stopping rule were pre-registered at https://osf.io/bz6c2/register/5771ca429ad5a1020de2872e. This left 68 participants (aged 18–70, mean 42) in the final analysis. The materials are the same as those from Experiment 1.

The procedure was the same as in Experiment 1, except that the task for the participants in Experiment 2 was to rate the consistency between what they heard and what read based on a 1–6 scale. 1 stood for *completely inconsistent*, while 6 stood for *completely consistent*. The participants were guided to go through two practice trials: one practice is an example of *completely inconsistent*, while the other is a practice of *completely consistent*, before starting the experiment. The example of *completely consistent* is provided in (6), where the audio content emphasized the quantity of water and has no conflicts with the written content.

(6) Written content: *Huang laoshi tongchang he henduo sui.Ta jintian hen mang. Ta mei he sui ye mei he kele. Ta de xuesheng gen ni shuo:* ‘Mr. Huang usually drank a lot of water. He was very busy today. He drank neither water nor coke. His student said:’
  Audio content: *Ta* ***yi di*** *sui dou mei he*. ‘He didn’t eat even **one drop of** water.’

#### Results and Discussion

The full dataset and analysis code are available at https://osf.io/nsgfv/.

**FIGURE 2:**
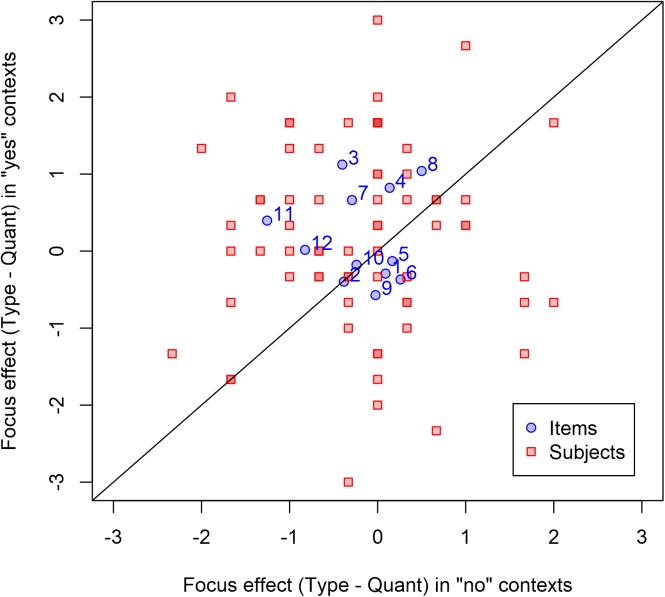
Effects of prosody in Experiment 2. Blue points represent items and red squares represent subjects; as in Experiment 1, points below the diagonal are subjects or items with differences in the predicted direction. Note that at several places there are multiple subjects with points in the same location; these can be recognized by the darker square backgrounds (since the background coloring is opaque). For clarity, point labels are provided only for items, not for subjects.

In contexts where the alternative type is present, the mean consistency rating was 4.9 for sentences with noun-stress prosody and 4.7 for sentences with quantity-stress prosody; this difference is opposite the predicted direction. In contexts where the alternative type is absent, consistency ratings were 5.2 for noun-stress prosody and 5.4 for quantity-stress prosody. In both contexts, the mean consistency rating was fairly high. The distribution of differences by subjects and items is shown in Figure 2. Since the effects were opposite the predicted direction, inferential statistics were not conducted.

Experiment 2 failed to replicate the trend observed in Experiment 1. We suspected that the effects of prosody may have been weakened or obscured in these experiments by two factors. First, the experimental contexts did not particularly draw participants’ attention to prosody, and in fact may have drawn their attention more to lexico-semantic factors. Since the experiment included fillers in which the target sentence clearly mismatched the context based on basic semantics as in (7), many participants’ attention may have been focused more on these issues. This type of filler may have become the standard for *completely inconsistent* for participants. Therefore, participants may have considered sentences with inconsistent prosody but consistent semantics to be fairly acceptable by comparison.

(7) Reading content: *Wangfang shi chuan le liang jian yifu, zuihou meiyou mai_∘_Dianyuan gen ni shou:* ‘Fang Wang tried on two pieces of clothes. She didn’t buy any. The shop assistant told you:’
  Audio content: *Ta yi jian yifu dou mei shi chuan*. ‘She didn’t try on even one piece of clothes.’

Secondly, Experiments 1 and 2 tested the effects of prosody on inferences indirectly, by testing whether prosody engenders an inference which mismatches a context (rather than by directly testing whether prosody engenders a given inference at all). In these two experiments, both prosody and lexico-semantic contents might influence the participants’ judgments. Thus, the results are not merely reflective of prosody. In Experiment 3 we attempted to address these issues by using a more direct approach, and by using a design meant to explicitly draw participants’ attention to prosody.

### Experiment 3: Comparing Types of Intonation

The experiment is designed to force participants to focus on prosody by providing different prosodic patterns and minimizing contextual information. In this experiment, participants had to listen to two sentences which differ in intonation. Afterward, they had to choose which sentence matched the provided context.

#### Participants, Materials, and Procedure

Sixty-four native speakers of Mandarin (63 users of traditional Chinese characters, 1 user of simplified Chinese characters) attended this experiment. Eleven were excluded for answering baseline questions incorrectly, and four for having low accuracy in the unambiguous filler trials. This left 49 participants (aged 18–60, mean 26) in the final analysis.

The experiment consists of 12 critical sentences along with 6 fillers. The critical sentences are in the format of (2). The participants were asked to listen to the same sentence in two kinds of prosodic patterns: one with stress on the noun (e.g., *In the cat café I didn’t see even one* ***cat***), one with stress on the numeral-classifier combination (e.g., *In the cat café I didn’t see even* ***one*** *cat*). Afterward, they were asked to choose the most appropriate answer to be the first clause of a two-clause sentence. The question appears in the format as (8)a or (8)b. (8)a provides an alternative in the category of types, whereas (8)b offers an alternative in the domain of quantity.

(8) (a)_____, gengbieshuo you guke le
  much less there be customer ASP
  ‘___, much less customers.’ [an alternative in type]
  (b) _____, gengbieshuo you yi qun maomi le
    much less there be one CLF cat ASP
    ‘___, much less a group of cats.’ [an alternative in quantity]

This version of the experiment only had two conditions: follow-up contexts which stress the type alternative, and follow-up contexts which stress the quantity alternative. We predicted that sentences with stress on the noun (consistent with type focus) would be selected more often when the follow-up sentence stresses the type alternative (8)a than when it stresses the quantity alternative (8)b. The items were organized into two lists in a Latin square design. There were six fillers, which also appear in the same format.

Among the fillers, three of them were in positive polarity environments, and three of them are in negative polarity environments. For each trial, two audio files were provided: one option matches the follow-up context (9)a, while the other mismatches the follow-up context (9)b.

(9) ___________, *genbieshuo xiao gongyu le*.
  ‘_______, much less a small apartment.’
  Audio files:
  (a) Match: *Ta mai de qi chengshi li de da haozhai*…. ‘He can afford a mansion in the city…’
  (b) Mismatch: *Ta chi de qi niupai*… ‘He can afford steaks…’

The fillers, which were unambiguous, also served to check the validity of the responses. The full list of stimuli is available at https://osf.io/nsgfv/.

This experiment was administered via Ibex Farm. The 12 critical items along with 6 fillers were presented in a fully random order after two practice trials. The practices were designed to direct participants’ attention to prosodic differences. As in (10), the two audio files have the same format, but the placement of a contrastive stress determined the item to be contrasted. According to the written context provided in (10), only the prosody of (10)a can match the follow-up sentence.

(10) ___________, *bu shi Xiaohan hui*.
  ‘___________, not Xiaohan who is able to.’
  Audio files:
  (a) *wo zhidao* ***ta*** *hui tiaowu* ‘I know it is she who is able to dance.’ [contrastive stress on *ta* ‘she’]
  (b) *wo zhidao ta* ***hui tiaowu*** ‘I know it is cooking that she is able to do.’ [contrastive stress on *hui tiaowu* ‘be able to dance’]

For each trial, with a written context sentence and two audio clips occurred on the screen at the same time. The task for the participants is to choose one of two audio clips to complete the sentence shown on the screen, which only the second clause of a two-clause sentence is provided. Participants could play the audio clips more than one time. The self-paced survey took less than 30 min to finish.

#### Results

The data and analysis code are available at https://osf.io/nsgfv/.

**FIGURE 3:**
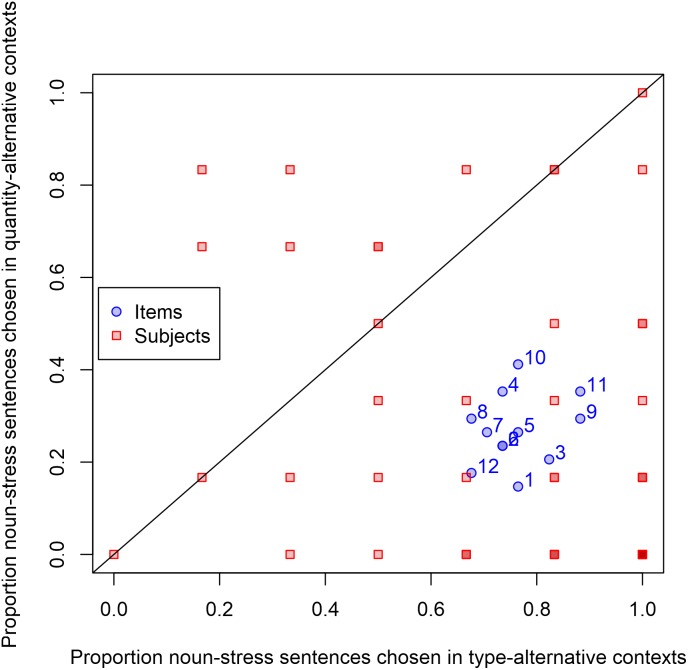
Effects of context in Experiment 3. Each point represents, for a given stimulus item or participant, the proportion of trials in which the sentence with stress on the noun was selected. Since we predicted more selection of noun-stress sentences in contexts evoking the type alternative than in contexts evoking the quantity alternative, that means the prediction is that the mean should be below the diagonal line.

As shown in Figure 3, sentences with noun stress were chosen more often in contexts that evoked the type alternative than in contexts that evoked the quantity alternative; conversely, sentences with quantifier stress were chosen more often in contexts that evoked the quantity alternative than contexts that evoked the type alternative.

The context effect was analyzed with a generalized (binomial) mixed-effects model regressing the binary response (coded with quantifier stress as the baseline level) on the fixed effect of context (dummy-coded with quantity-alternative contexts as the baseline level) and maximal random effects for subjects and items. This model revealed a significant effect of context (*b* = 358, *z* = 5.83, *p* < 0.001), indicating that the likelihood of selecting a sentence with stress on the noun was significantly higher in type-alternative contexts than in quantity-alternative contexts.

## General Discussion

This study tested whether prosody plays a role in pragmatic judgments, especially in terms of differentiating interpretational ambiguity of scalar implicatures. The connections among focus, prosody, and pragmatic inferences have been hinted in different literature (Chao, 1968; Haspelmath, 1997; Zhang, 2000; Tsai, 2004; Lee, 2006; Nakanishi, 2006; Israel, 2011), and prosody is known to influence utterance interpretation in other kinds of structures (e.g., Snedeker and Trueswell, 2003; Chevallier et al., 2010; Degen and Tanenhaus, 2015; among others), but the relations have not yet been specified for ambiguous alternative sets invoked by minimizers. In order to find empirical evidence for such an influence, we tested whether the type-contrast and quantity-contrast prosodic patterns can guide Mandarin native speakers to the correspondent scalar inferences. The results of the experiments suggest that Mandarin native speakers may use prosody to inform their interpretations of minimizers, but not necessarily in all contexts. The stimuli appear in the same syntactic structure, which has the numeral ‘one’ and the classifier specified. This syntactic pattern inherently entails the semantics of quantity. According to the participants’ responses, they tend to use the quantity contrast for this syntactic structure if the experiment design does not strongly draw their awareness to the prosodic changes. The expected effects of prosody were not strong in Experiment 1, and not present at all in Experiment 2; this may have been because in this setting the participants’ attention was drawn to syntax and semantics than to prosody. In this case, the participants judged the consistency between what they heard and what they read based on the quantity-contrast inferences. The pattern of results in the first two experiments also suggests that this minimizer structure, with the quantity specified, strong prefer a quantity-contrast interpretation. However, when the role or prosody was tested in a design that more directly addressed alternative interpretations of the minimizer and that draw participants’ attention more explicitly to prosody as in Experiment 3, then participants’ judgments of scalar inferences were heavily influenced by the patterns of prosody, in the direction we had predicted. This suggests that prosody is a factor which Mandarin speakers use to identify alternative sets when interpreting minimizers. These results suggest that prosody has an influence not only on structural disambiguation (in cases where utterances may be parsed into multiple syntactic or semantic structures) and the choice of whether to apply an implicature at all (in cases where utterances may be interpreted with or without a conversational implicature), but also on what alternatives the same implicature operates over.

The experiments also provide the evidence for the observed connection between prosodic stress and minimizers in the literature. The occurrence of a prosodic stress contributes to inducing a set of pragmatic inferences coherent with contexts. The placement of a prosodic stress is an indicator of where the attention of the native Mandarin speakers would be. This relation between prosodic prominence and loci of attention helps to account for the concept of focus in the syntax in Mandarin Chinese.

## Conclusion

The present study provided evidence that different prosodic patterns can guide hearers to induce different scalar reasoning. It has been observed that ‘one’-phrases minimizers in numeral-classifier languages have two types of scalar inferences due to the structure as a numeral phrase (Lee, 2006; Nakanishi, 2006). The two types of inferences, quantity-contrast and type-contrast, are reflected in morphology in other numeral-classifier languages, but not in Mandarin.

It has been noted that the numeral ‘one’ in Mandarin minimizers may bear a stress, but the actual loci of the stress and the purpose the stress were not specified. The experiments provide evidence that the locus of a prosodic stress can carry pragmatic information play a role in evoking alternatives during sentence comprehensions. In conditions where syntax, semantics, and morphology do not differentiate types of scalar inferences, prosody can help native Mandarin speakers to determine the entailed conceptual scale. However, according to the results of Experiments 1 and 2, the role of contextual information may sometimes override that of prosody in determining scalar inferences.

Although the placement of a prosodic stress specifies the types of inferences, the induced scalar inferences are asymmetrical as shown in the results of experiments. The quantity-contrast inferences involve choosing from a set of alternatives that are already lexically entailed by the minimizer. On the contrary, type-contrast inferences require choosing from an open set of nouns and an open set of conceptual scales, which is highly dependent on the context: i.e., there is no natural ordered ranking or entailment relationship between cats and people; in some contexts cats may be less likely than people (and the presence of cats may entail the presence of people), in other contexts the opposite may be true, and in still other contexts they may have no such relationships at all. Thus, the role of prosody in the contexts of type-contrasts is more difficult to test in a controlled fashion because it is difficult to predict which specific alternatives will be ruled out by this interpretation across different interlocutors and contexts.

In terms of our experimental results, what is the precise role of prosody in the processing of scalar implicatures in Mandarin Chinese? Note that Experiments 1 and 2 suggest that scalar implicatures are strongly defaulted to quantity type inferences regardless of the potential ambiguity. Hence Experiment 3 is the critical one that shows the effect of prosody on interpretation and the effect is the over-riding of the default. As there is no reason to believe that the prosodic stress directly encodes either interpretation, a likely explanation is that stress brings attention to a typically less likely interpretation, such as flagging or underlining parts of a text. It is possible that type-contrast is more cognitively costly to realize, as it requires generating a context-dependent set of alternatives, as opposed to the lexically-encoded set of alternatives (i.e., “not one” entails “not two,” “not three,” “not four,” etc.) used for quantity-contrast. If that is the case, participants may avoid realizing a type contrast unless either the contrast is made less cognitively costly [e.g., if specific alternatives are made salient in the preceding contrast; relatedly, experiments have suggested that *ad hoc* scalar implicatures can be realized with little processing cost if the ad-hoc scale is already contextually salient (Breheny et al., 2013; Politzer-Ahles and Fiorentino, 2013)] or if additional cues give them evidence that this contrast is particularly relevant and thus worth the effort. If this is the case, the prosodic cues may act to trigger the additional processing of potential type inferences.

The experiments of this study show that prosody can play a role in influencing the kind of scalar inferences that are induced by a minimizer in Mandarin. The prosodic conditions are considered when the non-default type inference needs to be processed. Hence, the effects of prosody on determining types of scalar inferences can be diminished by contextual information. The types of scalar inferences in Mandarin are determined by how prosody and contextual information interact.

## Ethics Statement

This study was carried out in accordance with the recommendations of Hong Kong Polytechnic University, Human Subjects Ethics Sub-committee. The protocol was approved by the Hong Kong Polytechnic University, Human Subjects Ethics Sub-committee. All subjects gave written informed consent in accordance with the Declaration of Helsinki.

## Author Contributions

I-HC and SP-A contributed to conception and design of this study. I-HC executed the experiments and analyzed the results. SP-A advised the design of experiments, supervised the procedure of experiments, and performed the statistical analysis. C-RH contributed to the linguistic theory and interpretation of the results of this study. I-HC wrote the first draft of the manuscript. All authors contributed to manuscript revision and approved the submitted version.

## Conflict of Interest Statement

The authors declare that the research was conducted in the absence of any commercial or financial relationships that could be construed as a potential conflict of interest.
